# Spectrum of BRAF Aberrations and Its Potential Clinical Implications: Insights From Integrative Pan-Cancer Analysis

**DOI:** 10.3389/fbioe.2022.806851

**Published:** 2022-07-14

**Authors:** Qiaoli Yi, Jinwu Peng, Zhijie Xu, Qiuju Liang, Yuan Cai, Bi Peng, Qingchun He, Yuanliang Yan

**Affiliations:** ^1^ Department of Pharmacy, Xiangya Hospital, Central South University, Changsha, China; ^2^ Department of Pathology, Xiangya Hospital, Central South University, Changsha, China; ^3^ Department of Pathology, Xiangya Changde Hospital, Changde, China; ^4^ National Clinical Research Center for Geriatric Disorders, Xiangya Hospital, Central South University, Changsha, China; ^5^ Department of Emergency, Xiangya Hospital, Central South University, Changsha, China; ^6^ Department of Emergency, Xiangya Changde Hospital, Changde, China

**Keywords:** BRAF, gene fusion, alteration, prognosis, pan-cancer

## Abstract

B-Raf proto-oncogene serine/threonine-protein kinase (BRAF) is frequently altered in multiple cancer types, and BRAF V600 mutations act as a prime target for precision therapy. Although emerging evidence has investigated the role of BRAF, the comprehensive profiling of BRAF expression, alteration and clinical implications across various cancer types has not been reported. In this study, we used the TCGA dataset, covering 10,967 tumor samples across 32 cancer types, to analyze BRAF abnormal expression, DNA methylation, alterations (mutations and amplification/deletion), and their associations with patient survival. The results showed that BRAF expression, alteration frequency, mutation site distribution, and DNA methylation patterns varied tremendously among different cancer types. The expression of BRAF was found higher in PCPG and CHOL, and lower in TGCT and UCS compared to normal tissues. In terms of pathological stages, BRAF expression was significantly differentially expressed in COAD, KIRC, LUSC, and OV. The methylation levels of BRAF were significantly lower in LUSC, HNSC, and UCEC compared to normal tissue. The expression of BRAF and downstream gene (ETS2) was negatively correlated with methylation levels in various cancers. The overall somatic mutation frequency of BRAF was 7.7% for all cancer samples. Most fusion transcripts were found in THCA and SKCM with distinct fusion patterns. The majority of BRAF mutations were oncogenic and mainly distributed in the Pkinase_Tyr domain of THCA, SKCM, COADREAD, and LUAD. The BRAF mutations were divided into five levels according to the clinical targeted therapy implication. The results showed level 1 was mainly distributed in SKCM, COADREAD, and LUAD, while level 3B in THCA. The overall BRAF CNV frequency was about 42.7%, most of which was gain (75.9%), common in GBM, TGCT, and KIRP. In addition, the forest plot showed that increased BRAF expression was associated with poor patient overall survival in LIHC, OV, and UCEC. Taken together, this study provided a novel insight into the full alteration spectrum of BRAF and its implications for treatment and prognosis.

## Introduction

The B-Raf proto-oncogene serine/threonine-protein kinase (BRAF) is located on chromosome 7q34 and encodes a protein which belongs to the RAF family (ARAF, BRAF, and CRAF) of serine/threonine protein kinases. As a direct downstream effector of RAS, BRAF protein plays an important role in regulating the mitogen-activated protein kinase (MAPK)/extracellular signal-regulated kinase (ERK) signaling pathway, which mediates a variety of essential cellular processes, including cell growth, proliferation, differentiation and survival (Ritterhouse and Barletta, 2015; Song et al., 2020; Xu et al., 2020).

Mutations in BRAF, most frequently the valine (V) to glutamate (E) substitution at residue 600 (V600E), are identified as cancer-causing mutations in thyroid carcinoma (THCA) and skin cutaneous melanoma (SKCM). As an oncogenic driver, BRAF V600 mutations account for approximately 60% of all BRAF mutations in cancer patients. In contrast to wild-type BRAF, the constitutively active BRAF V600E mutation dramatically enhances kinase activity in an RAS-independent manner and is independent of protein homodimerization to switch to the highly active state (Samatar and Poulikakos, 2014; Alos et al., 2020). Up to now, the second-generation BRAF inhibitors vemurafenib (PLX4032) and dabrafenib (GSK2118436) were approved by the United States Food and Drug Administration (FDA) for the treatment of patients with metastatic melanomas harboring BRAF V600 mutations (Karoulia et al., 2017). Point mutations are not the only alterations found in BRAF. Fusion transcripts arising from translocations have been identified in melanoma, prostate cancer, gastric cancer, etc. (Palanisamy et al., 2010). The oncogenic potential of BRAF fusions has been attributed to the lack of a crucial N-terminal domain that mediates BRAF autoinhibition. Owing to the deletion of the N-terminal inhibitory domain, gene fusions lead to constitutive dimerization of BRAF protein aberrantly activating the downstream MAPK signaling pathway (Cremolini et al., 2019; Kratz and Deming, 2019).

Since previous studies of BRAF gene alterations in cancers are limited to a single cancer type and/or insufficient sample sizes, an integrative analysis across a variety of tumor types to investigate its function is of particular importance. In this article, we comprehensively analyzed the large dataset from The Cancer Genome Atlas (TCGA) to fill in the gaps. We first systematically profiled BRAF expression, methylation, gene alterations, and its clinical and therapeutic implications across 32 TCGA cancer types covering 10,967 tumor samples. In addition, the survival associations between BRAF expression and prognosis in distinct cancer types were conducted to explore its potential therapeutic implication. In general, our study provided a novel insight into the full alteration spectrum of BRAF and its implications for treatment and prognosis in diverse tumor types.

## Materials and Methods

### Data Acquisition and Reanalysis Using Bioinformatics Tools

The essential bioinformatics tools used in this research could be found in Supplementary Table S1. Tumor Immune Estimation Resource (TIMER2.0) is a comprehensive resource for the systematical analysis of immune infiltrates across diverse cancer types (Li et al., 2020). We studied the differential expression of the BRAF gene between tumor samples and adjacent normal tissues across all TCGA tumors by using the “Gene_DE” module of the TIMER2.0 database. The transcripts per million (TPM) values of transcription factors were log2-converted. For certain cancer types without adjacent normal tissues, we further explored Gene Expression Profiling Interactive Analysis 2 (GEPIA2) portal to investigate the BRAF mRNA expression difference between tumor samples and matched TCGA normal and Genotype-Tissue Expression (GTEx) data. Additionally, the GEPIA2 data portal was also used to generate violin plots of BRAF expression across pathological stages for all TCGA cancer types. The log2 (TPM +1) transformed expression data were applied for the violin plots here. GEPIA2 is an interactive web server for analyzing the RNA sequencing expression data from the TCGA and the GTEx projects and provides customizable functions such as tumor/normal differential expression analysis, survival analysis, and so on (Tang et al., 2019). Next, we analyzed the methylation difference of BRAF and its downstream genes of the MAPK signaling pathway between tumor samples and adjacent normal tissues in various TCGA cancer types by using the “TCGA Cancer-Methylation” module in the Gene Set Cancer Analysis (GSCALite) platform. Furthermore, the correlation between methylation and gene expression of BRAF and downstream genes was also visualized by the GSCALite platform. GSCALite is a web-based analysis platform that integrates cancer genomics data to analyze gene methylation, drug sensitivity, and so on (Liu et al., 2018).

The cBioPortal is an open-access portal that provides an interactive investigation of multidimensional cancer genomics and clinical data (Gao et al., 2013). In this study, we selected the “TCGA PanCancer Atlas Studies” covering 10,967 samples across 32 cancer types to further explore BRAF alterations. Data files including copy-number alterations, mutations, mRNA Expression, Log2 copy-number values (CNV), and clinical data were downloaded from cBioportal. The BRAF mRNA expression data were performed based on RSEM (batch normalized from Illumina HiSeq_RNASeqV2) and then log10 transformed. For the BRAF CNV data, the log-ratio value represents: 2 = amplification; 1 = gain; 0 = diploid; -1 = shallow deletion; and −2 = deep deletion. In addition, BRAF fusion data were collected from the TCGA Fusion Gene Database, which enables researchers to query cancer-associated transcript fusions in an interactive manner (Hu et al., 2018).

The Kaplan-Meier plotter is an open-access online database that enables researchers to assess the effect of a candidate gene on survival analysis in pan-cancer (Gyorffy, 2021). The correlations between BRAF mRNA expression and clinical prognosis across various cancer types were analyzed with the “Start KM Plotter for pan-cancer” module. Data including hazard ratio (HR), *p*-value, and 95% confidence interval (CI) were collected to draw the forest plots to summarize survival analysis.

### Statistical Analyses

The statistical analysis was performed with Graphpad PRISM software Version 8.0. Student’s t-test, Cox regression analysis, and linear regression analysis were conducted when appropriate. *p* < 0.05 was defined as a statistically significant difference.

## Results

### Expression and Methylation Level of BRAF in Pan-Cancer

**FIGURE 1:**
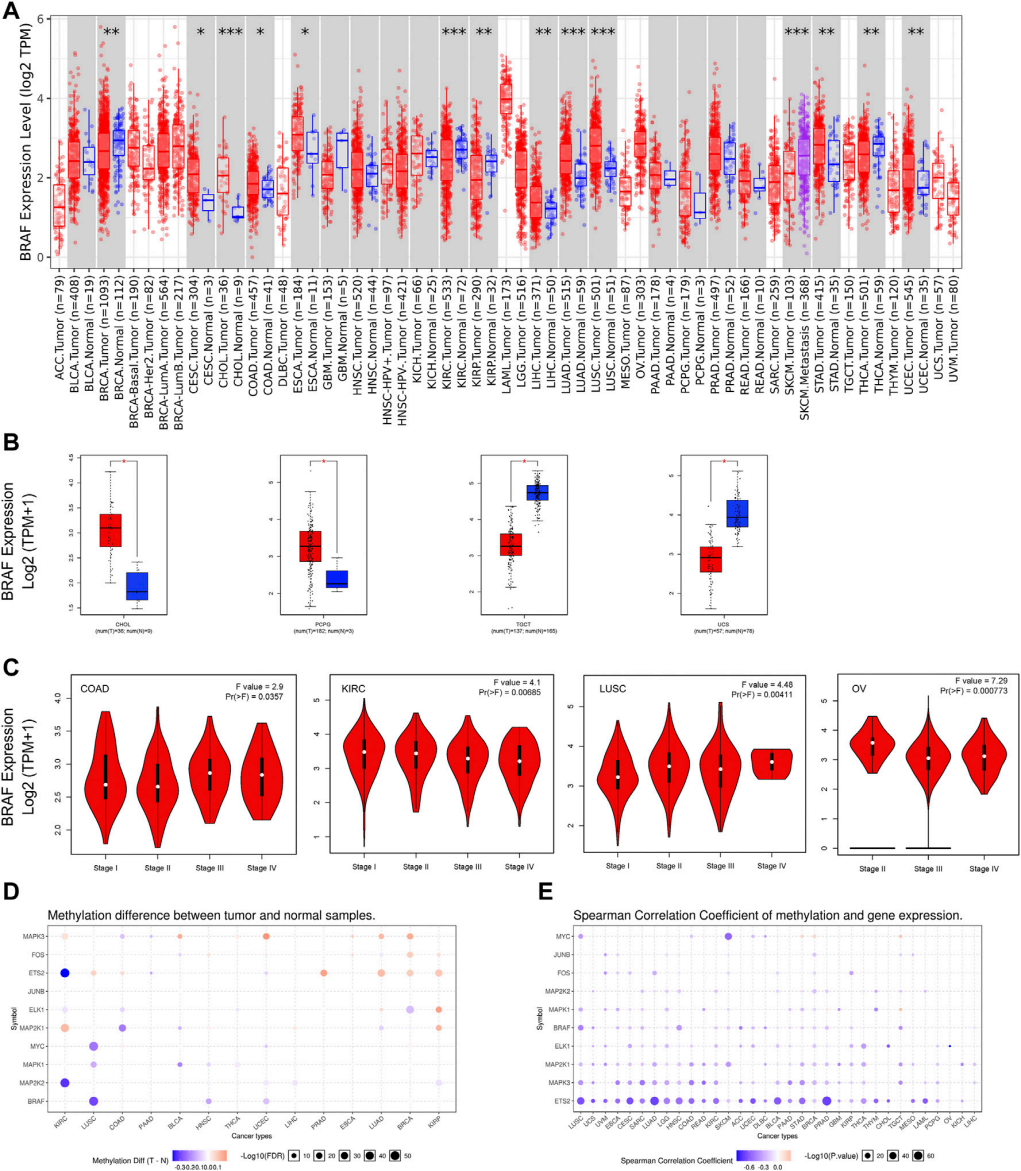
BRAF mRNA expression and DNA methylation in TCGA tumor tissues. **(A)** BRAF mRNA expression across different cancer types by TIMER2. The log2 [TPM (Transcripts per million)] was applied for the log-scale. **(B)** Differential expression of BRAF between tumors samples and normal tissues using combined data from TCGA and GTEx datasets based on the GEPIA2 portal. BRAF expression was up-regulated in CHOL and PCPG, but down-regulated in TGCT and UCS. The log2 (TPM + 1) was applied for log-scale. **(C)** Differential expression of BRAF in different pathological stages of COAD, KIRC, LUSC, and OV. The log2 (TPM + 1) was applied for log-scale. **(D)** Bubble map depicting the methylation difference of BRAF and its downstream genes between tumors and normal samples. Blue dots indicate down-regulated methylation in tumors. Red dots indicate up-regulated methylation. **(E)** Bubble map exhibiting correlations between methylation and gene expression of BRAF and its downstream genes. Blue dots denote down-regulated methylation in tumors. Blue dots represent the negative Spearman correlation coefficient, and red dots represent the positive. *p < 0.05; **p < 0.01; ***p < 0.001.

Aberrant expression of BRAF gene has been demonstrated in various cancer types (Wang et al., 2021; Zhao et al., 2021). Previous studies on BRAF expression in cancer have used inconsistent research methods and have been limited to small sample sizes and/or to single or limited cancer types. In this study, we conducted a more comprehensive analysis of BRAF expression in pan-cancer. At the outset, we explored the mRNA expression pattern of BRAF between tumor samples and adjacent normal tissues in pan-cancer by TIMER2.0 (Figure 1A). Compared with the corresponding adjacent normal tissues or metastatic lesions, significantly differential expression of BRAF was found in 14 cancer types, with 9 tumor types up-regulated (cervical squamous cell carcinoma and endocervical adenocarcinoma (CESC), cholangiocarcinoma (CHOL), colon adenocarcinoma (COAD), esophageal carcinoma (ESCA), liver hepatocellular carcinoma (LIHC), lung adenocarcinoma (LUAD), lung squamous cell carcinoma (LUSC), stomach adenocarcinoma (STAD) and uterine corpus endometrial carcinoma (UCEC)) and 5 tumor types down-regulated (breast invasive carcinoma (BRCA), kidney renal clear cell carcinoma (KIRC), kidney renal papillary cell carcinoma (KIRP), SKCM, THCA). After adding GTEx normal tissue samples as a control, we further investigated BRAF differential expression by GEPIA2. As shown in Figure 1B, BRAF expression was up-regulated in pheochromocytoma and paraganglioma (PCPG) and CHOL, down-regulated in testicular germ cell tumors (TGCT) and uterine carcinosarcoma (UCS). We further analyzed the correlation between BRAF expression and pathological stages in pan-cancer. As shown in Figure 1C, we found that BRAF expression was correlated with pathological stages in several tumor types, including COAD, KIRC, LUSC, and ovarian serous cystadenocarcinoma (OV) (*p* < 0.05).

A growing body of evidence has suggested that DNA methylation is strongly correlated with gene alteration in cancers (Wang et al., 2020; Zhang et al., 2020). Therefore, we searched the methylation profiles of BRAF and its downstream genes in TCGA cancers by using the GSCALite database (Figures 1D,E). The results indicated that the methylation of BRAF was down-regulated in LUSC, head and neck squamous cell carcinoma (HNSC), and UCEC. Then, we evaluated the correlation between methylation and BRAF expression in pan-cancer. The results revealed that the expression profiles of BRAF and downstream genes were generally negatively correlated with methylation in various cancers.

### BRAF Somatic Mutation Patterns in Pan-Cancer

**FIGURE 2:**
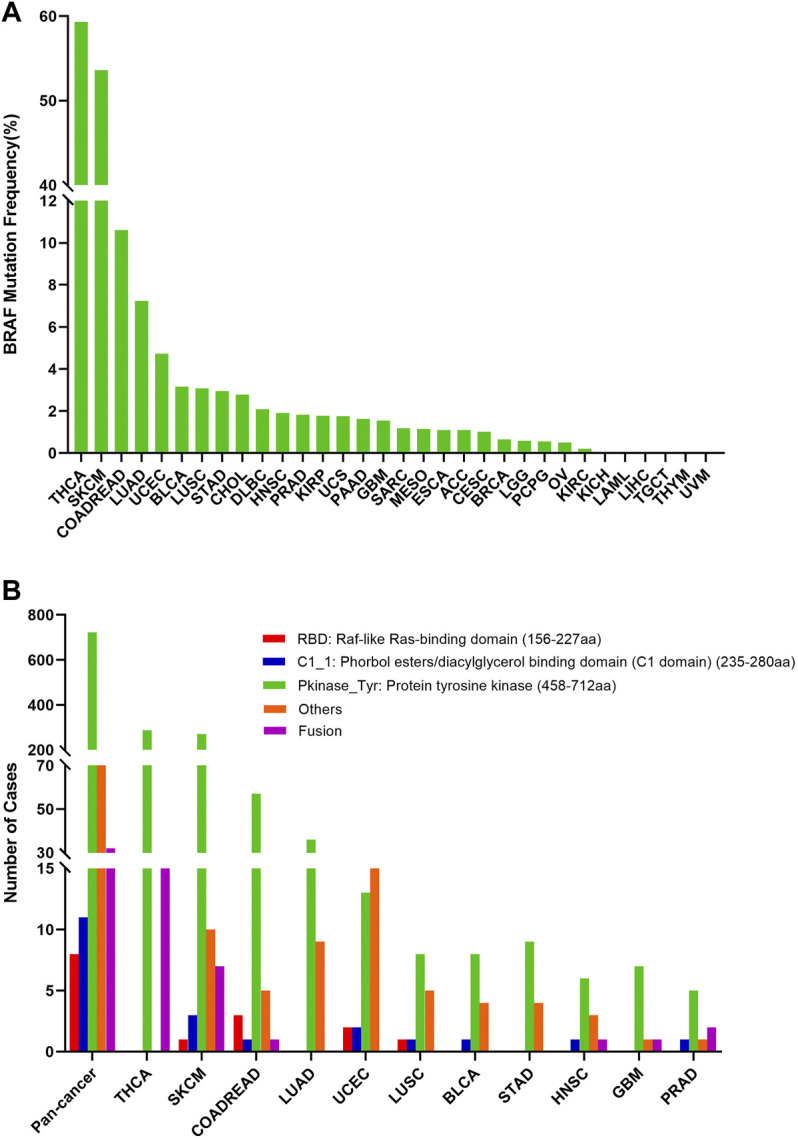
BRAF mutation distribution in various cancer types and protein functional domains. **(A)** BRAF mutation frequency across 32 TCGA cancer types. **(B)** BRAF mutation distribution in different protein functional domains in all and top 10 tumor types. Abbreviation: aa: amino acid.

The overall somatic mutation frequency of BRAF was 7.7% for all cancer samples (848/10,976) and 7.0% for all patients (767/10,953) across the 32 TCGA cancer types. And the detailed information on 848 BRAF somatic mutations was shown in Supplementary Table S2. The sample size of each tumor type varied from 36 (CHOL) to 1,084 (BRCA), and the cancer types with a small sample size might not reflect the general spectrum of BRAF mutation status (Supplementary Table S3). As shown in Figure 2A, the most frequent cancer types with BRAF mutations were THCA (59.3%), SKCM (53.6%), colon adenocarcinoma/rectum adenocarcinoma (COADREAD) (10.6%), LUAD (7.2%) and UCEC (4.7%). Instead, almost no BRAF mutations were observed in kidney chromophobe (KICH), acute myeloid leukemia (LAML), LIHC, TGCT, thymoma (THYM), and uveal melanoma (UVM).

Based on the Pfam database (http://pfam.xfam.org/protein/braf_human), BRAF harbors 3 functional domains, including the RBD domain (156-227 aa), C1_1 domain (235-280 aa), and Pkinase_Tyr domain (458-712 aa). The 848 BRAF somatic mutations were observed in various cancer types and widely distributed across different functional domains of the BRAF gene. The most common one was the Pkinase_Tyr domain (722 samples), followed by the other domains whose functions were barely known (75 samples), the C1_1 domain (11 samples), and the RBD domain (8 samples). Fusions (32 samples) were also observed in BRAF somatic mutations across all cancer types. The location distribution of BRAF mutations was dramatically different among numerous cancers. Mutations in THCA, SKCM, COADREAD, and LUAD were most frequently distributed in the Pkinase_Tyr domain. However, mutations in UCEC were predominantly located in the other domains amounting to half of the total mutations. Furthermore, fusions were mainly distributed in THCA and SKCM (Figure 2B and Supplementary Table S4).

**FIGURE 3:**
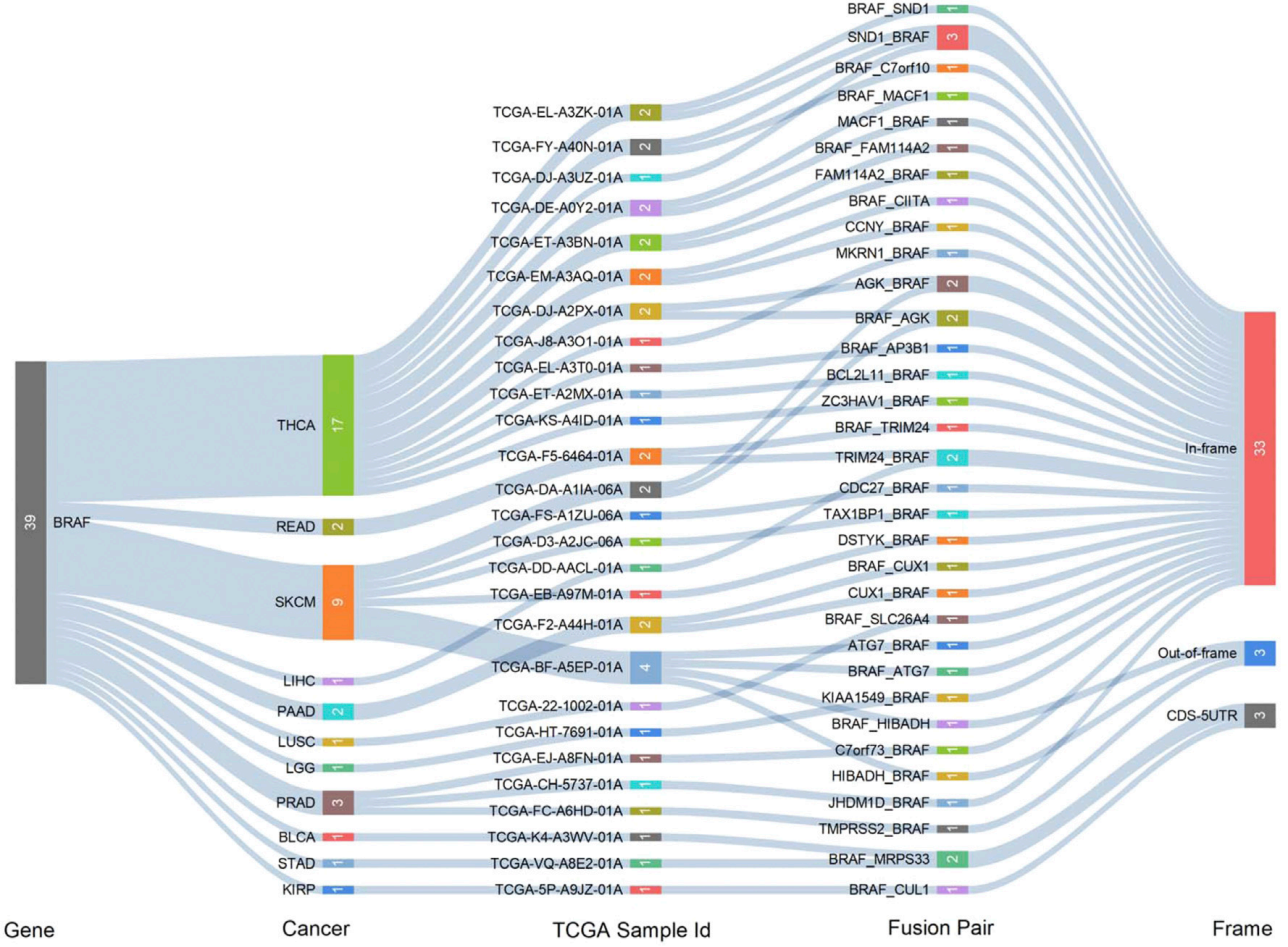
Gene fusions of BRAF across different cancer types.

Fusion genes generated by cleavage and re-splicing at the genome level are often the targets for tumor diagnosis and treatment. We analyzed fusion transcripts of BRAF across various cancer types by using the TCGA Fusion Gene Database (Figure 3). BRAF fusion transcripts were detected in THCA (17), SKCM (9), prostate adenocarcinoma (PRAD) (3), pancreatic adenocarcinoma (PAAD) (2), READ (2), LIHC (1), LUSC (1), STAD (1), KIRP (1), brain lower-grade glioma (LGG) (1) and bladder urothelial carcinoma (BLCA) (1). The highest number of fusion transcripts was found in THCA (three SND1_BRAF, one BRAF_SND1, one AGK_BRAF, one BRAF_AGK, one MACF1_BRAF, one BRAF_MACF1, one FAM114A2_BRAF, one BRAF_ FAM114A2, one CCNY_BRAF, one MKRN1_BRAF, etc.). AGK_BRAF and BRAF_AGK were also detected in SKCM. The vast majority of these BRAF fusion transcripts were classified as in-frame, while three BRAF fusion transcripts (one BRAF_HIBADH and one HIBADH_BRAF in SKCM, one TMPRSS2_BRAF in PRAD) were classified as out-of-frame and three (one BRAF_MRPS33 in BLCA and one in STAD, one BRAF_CUL1 in KIRP) were classified as CDS-5UTR.

According to functional impact on protein coding, these 848 BRAF somatic mutations could be classified into four categories: missense (778 mutations), truncating (32 mutations), fusion (32 samples), and in-frame (6 mutations) (Supplementary Figure S1A). The 600aa in the Pkinase_Tyr domain was the most mutated position, which was observed in 590 samples (545 samples with V600E, 39 with V600M, 4 with V600G, 1 with V600_K601delinsE and 1 with V600_R603del). Mutations at V600E were primarily distributed in THCA samples (284/545) and SKCM samples (193/545) (Supplementary Figure S1B–C). V600E is known to be oncogenic and serves as target for BRAF inhibitors approved by FDA, while other mutation types (V600M/G) are likely oncogenic. Other tumors with mutations at this position were COADREAD (48 samples), LUAD (9 samples), glioblastoma multiforme (GBM) (5 samples), KIRP (2 samples), BLCA (1 sample), LGG (1 sample), CHOL (1 sample) and HNSC (1 sample). Studies on their role in COADREAD and LUAD are underway (Planchard et al., 2017; Kopetz et al., 2019) and its function remains little known in other cancer types.

**FIGURE 4:**
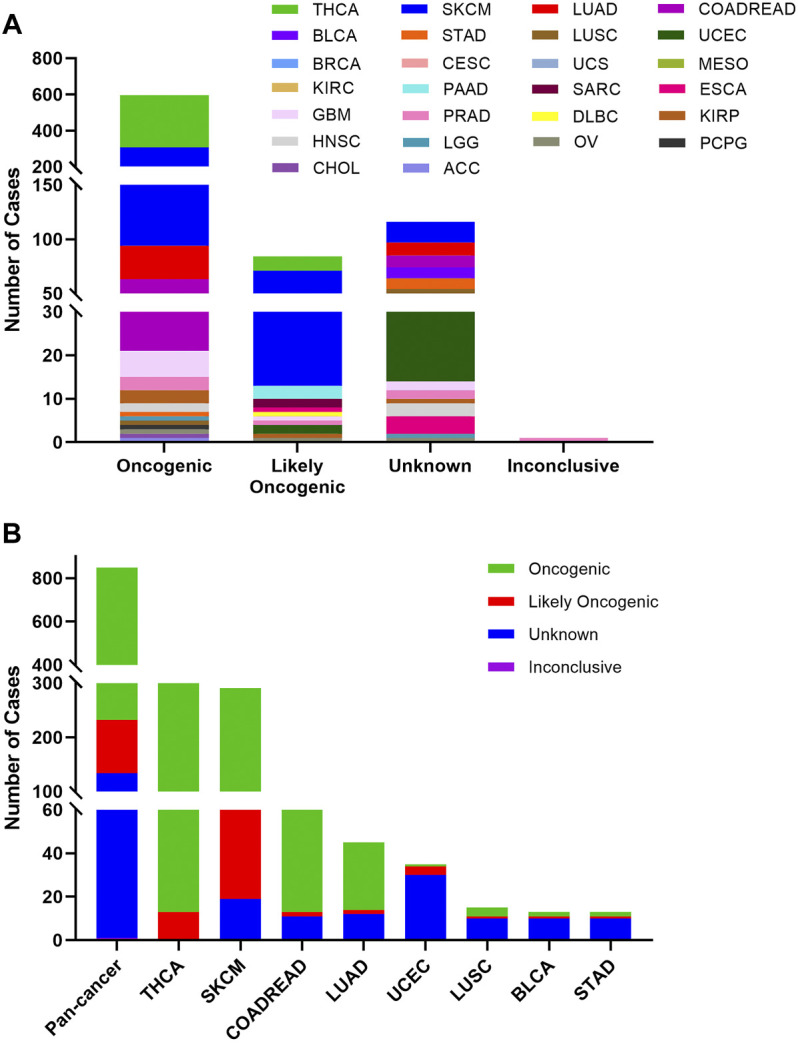
BRAF mutation classification by functional impacts. **(A)** BRAF mutations based on functional impacts on all tumors together. **(B)** Functional impact class distribution of BRAF mutations in pan-cancer and the top 8 cancer types.

Based on the oncogenic effect and predictive significance, the 848 BRAF somatic mutations could be classified into four categories. As shown in Figure 4A, 616 (72.6%) BRAF mutations were oncogenic, 98 (11.6%) likely oncogenic, 1 (0.1%) inconclusive and 133 (15.7%) unknown. Although a major portion of BRAF somatic mutations was distributed in the functional categories, there were still some mutations in the unknown class deserving further study to characterize the potential functional significances of these mutations. As displayed in Figure 4B, mutations distributed in the functional categories comprised the majority of BRAF mutations in several cancers such as THCA, SKCM, COADREAD, and LUAD. However, more than two-thirds of mutations belonged to the unknown class in UCEC, LUSC, BRCA, and STAD.

**FIGURE 5:**
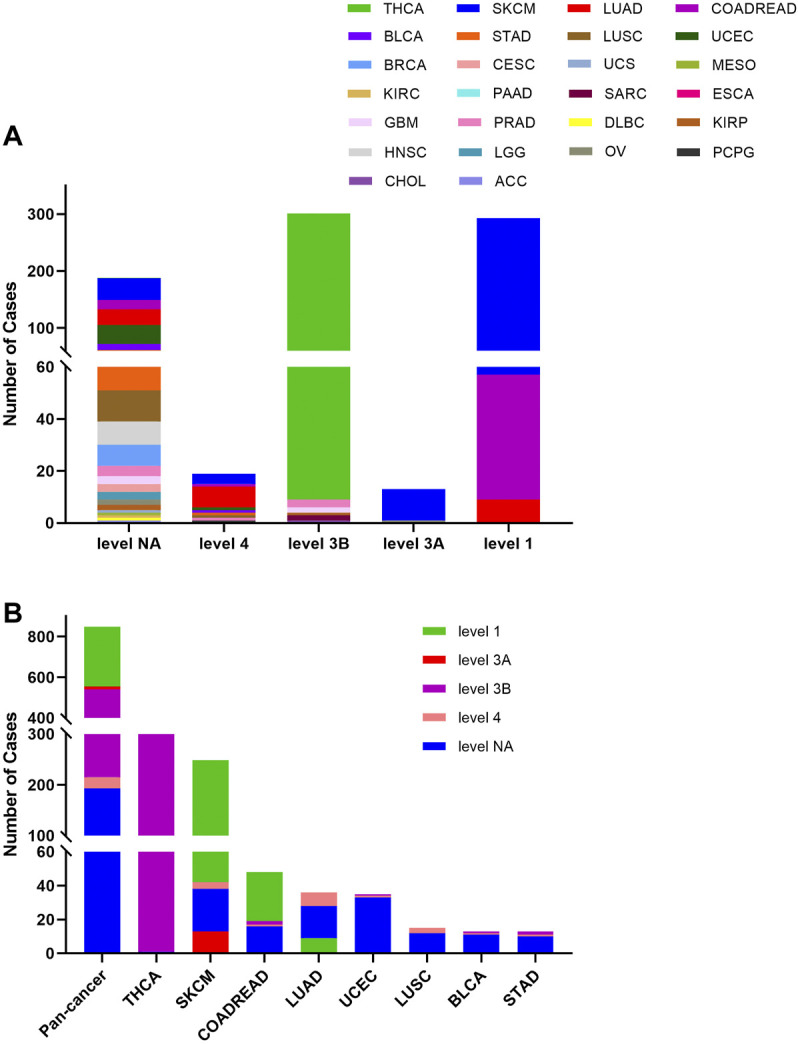
BRAF mutation distribution according to clinical therapeutic implications. **(A)** BRAF mutation distribution based on the OncoKB therapeutic evidence levels among diverse cancer types. **(B)** OncoKB therapeutic evidence levels distribution of BRAF mutation in pan-cancer and the top 8 tumor types.

The 848 BRAF mutations could be divided into five levels by the clinical targeted therapy implication, containing level NA (193 mutations), level 4 (22 mutations), level 3B (326 mutations), and level 3A (14 mutations), and level 1 (293 mutations). Only level 1 mutations are indicated for targeted therapy with FDA-approved drugs. All level 1 mutations were found in SKCM (236), COADREAD (48), and LUAD (9). These mutations (including V600E, V600M, and V600G) were concentrated in 600aa of the Pkinase_Tyr domain. Although all mutations in THCA belonged to oncogenic (289/302)/likely oncogenic (13/302), all of them were in level 3B (301/302) and level NA (1/302) without treatment implications (Figures 5A,B).

### BRAF CNVs in Different Tumor Types

**FIGURE 6:**
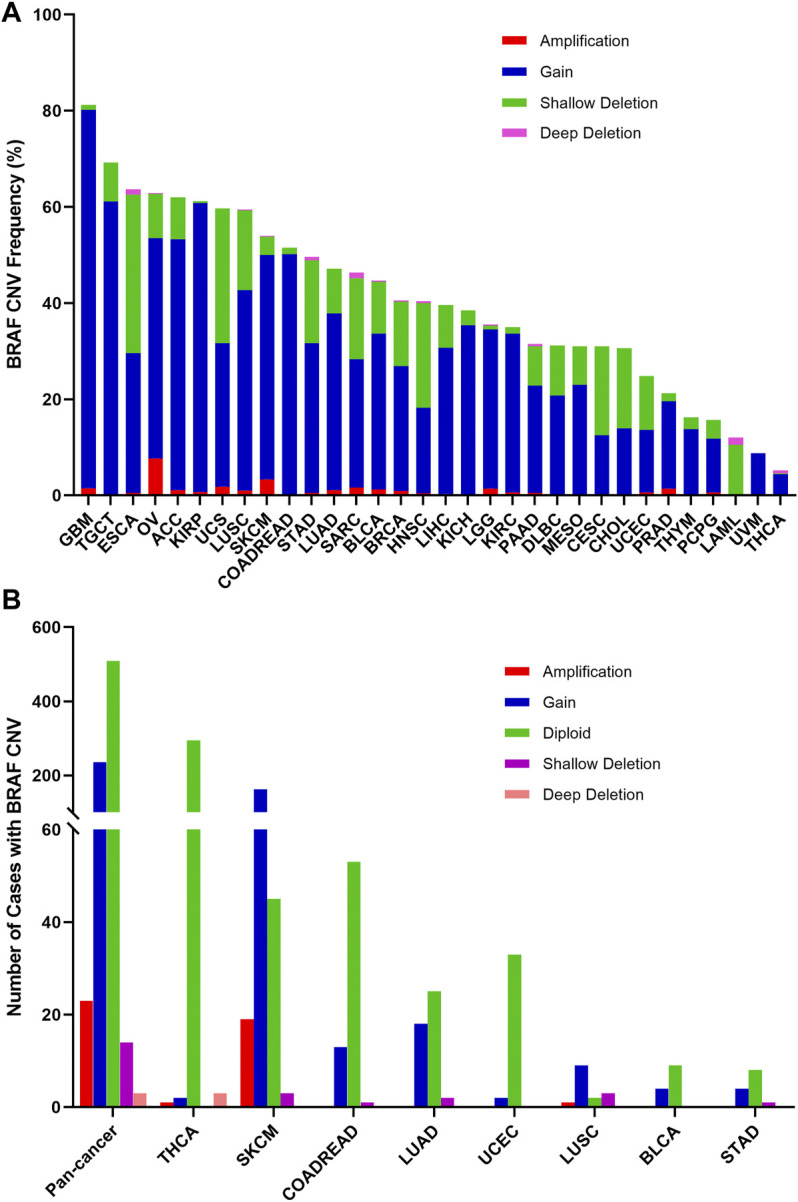
BRAF Copy Number Variant (CNV) distribution across all and selected top 8 cancer types. **(A)** BRAF CNV frequency in 32 TCGA cancer types. **(B)** BRAF CNV distribution in pan-cancer and the top 8 cancer types for the cases with EGFR mutations.

The overall BRAF CNV frequency was about 42.7% (4,684/10,967 samples). The majority of BRAF CNV types were gain (3,555 samples), followed by shallow deletion (972 samples), application (133 samples), and deep deletion (24 samples). The most common tumor types with BRAF CNVs were GBM (81.3%), TGCT (69.1%), ESCA (63.7%), OV (62.9%), adrenocortical carcinoma (ACC) (62.0%), KIRP (61.1%), UCS (59.6%) and LUSC (59.5%). By contrast, THYM (16.3%), PCPG (15.7%), LAML (12.0%), UVM (8.8%) and THCA (5.2%) harbored a relatively low BRAF CNV frequency (Figure 6A). Among the 848 samples with BRAF mutations described above, 276 also had BRAF CNV changes, of which 236 with gain, 23 with amplification, 14 with shallow deletion, and 3 with deep deletion. SKCM had the highest numbers of amplification or gain among different cancer types. Meanwhile, SKCM and LUSC were the two cancer types with the highest numbers of shallow deletion, and deep deletion only occurred in THCA (Figure 6B, Supplementary Table S2).

BRAF mRNA expression was compared across 32 TCGA cancer types and exhibited a relatively consistent trend, suggesting that there may be a common mechanism to promote BRAF expression. Based on the interquartile range, BRAF expression was widely distributed in COADREAD and SKCM, and narrowly distributed in UVM and mesothelioma (MESO), which may be attributed to the fact that some cancer types had more than one subtype and therefore more genetic diversity (Supplementary Figure 2A). In addition, we explored the correlation between BRAF mRNA expression and CNVs. The results showed that there was a positive correlation between BRAF mRNA expression and CNVs in pan-cancer (*r* = 0.1716, *p* < 0.0001) (Supplementary Figure 2B).

### Combined BRAF Alterations (CNVs and Mutation) Across Various Cancer Types

**FIGURE 7:**
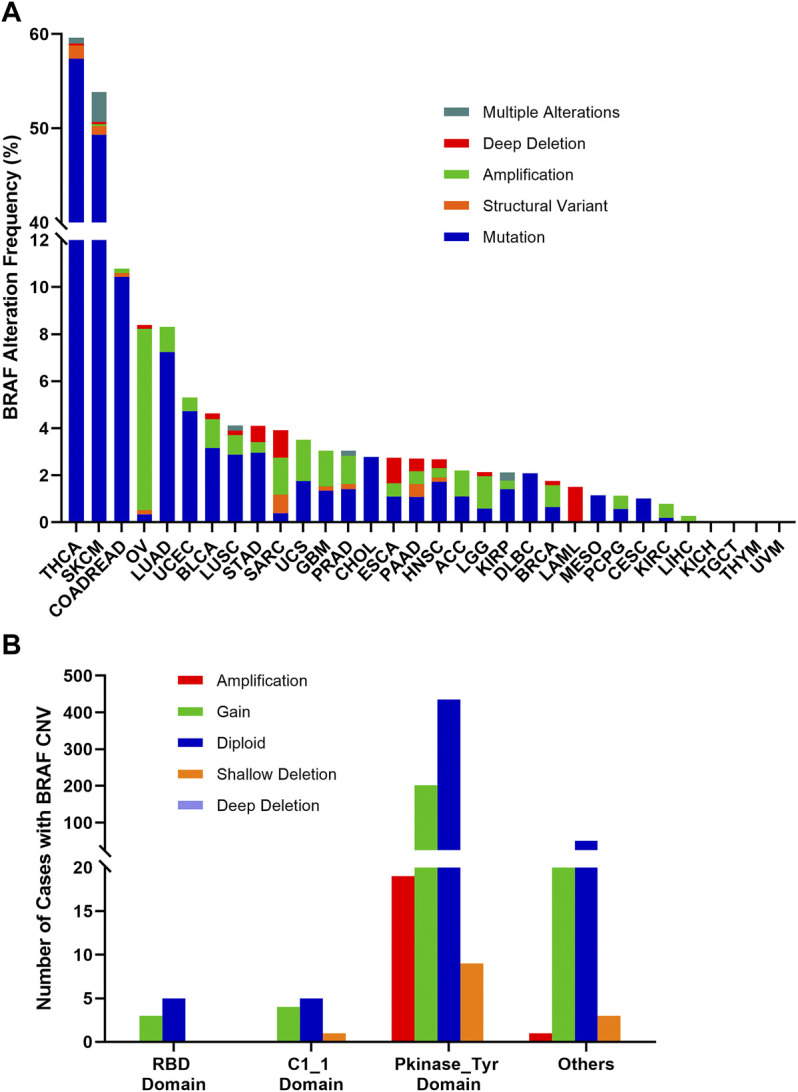
BRAF alterations and distribution in pan-cancer. **(A)** BRAF alteration (mutations and CNVs) frequency across 32 TCGA tumor types. **(B)** BRAF CNVs distribution along with mutations located in different protein functional domains.

The combined BRAF alteration (CNV and mutation) frequency in all cancers was about 8.3% (905 of 10,967 samples). As shown in Figure 7A, BRAF alteration frequency among various cancer types was dramatically different. While KICH, TGCT, THYM, and UVM had neither BRAF mutation nor BRAF CNVs, BRAF alterations were most frequently observed in THCA (59.6%) and SKCM (53.8%), in which mutation was more common than CNV, with BRAF mutation rates of 57.4 and 49.3%, respectively. Other cancers with dominant BRAF mutation but at much lower mutation rate included COADREAD (10.4%), LUAD (7.2%), UCEC (4.7%), BLCA (3.2%), STAD (3.0%), LUSC (2.9%), CHOL (2.8%) and DLBC (2.1%). Amplification was more common in OV (7.7 vs. 0.3%), SARC (1.6 vs. 0.4%) and LGG (1.4 vs. 0.6%). Deep deletion was mainly distributed in LAML (1.5%), SARC (1.2%) and ESCA (1.1%).

BRAF mutation location and its CNVs occurrence appeared to be associated. 201 of 664 (30.3%) mutations in the Pkinase_Tyr domain and 21 of 75 (28%) mutations in the other function-unknown domain were accompanied by copy gain. Amplification was mainly distributed in the Pkinase_Tyr domain. Mutations in the RBD domain and C1_1 domain rarely had concurrent CNVs (Figure 7B).

### BRAF Expression and the Prognosis of Cancer Patients

**FIGURE 8:**
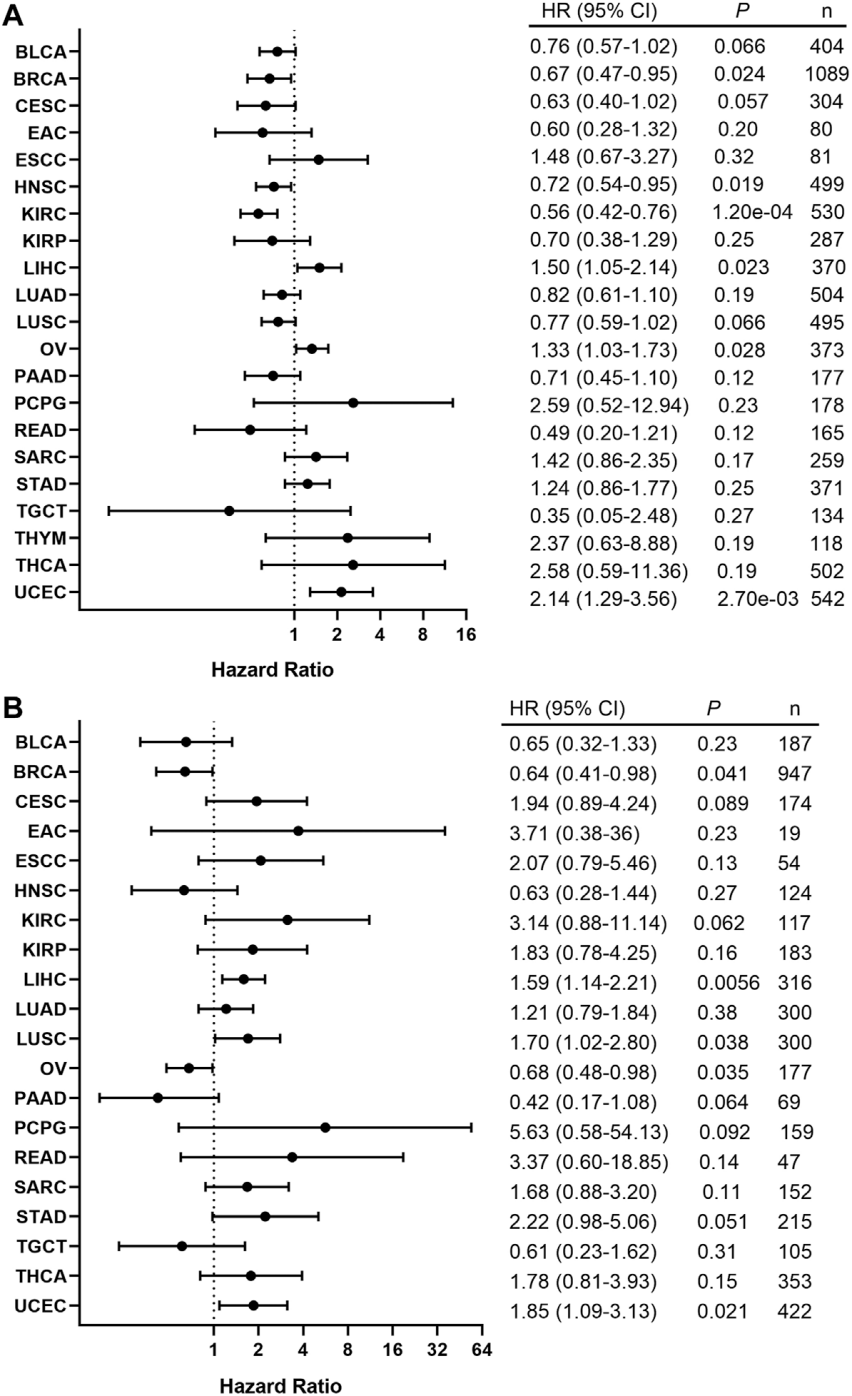
Correlation between the BRAF expression and patient survival. **(A)** Forest plot of the association between BRAF expression and overall survival (OS) based on Kaplan-Meier Plotter. **(B)** Forest plot of the association between BRAF expression and relapse-free survival (RFS) based on Kaplan-Meier Plotter.

In order to assess the clinical significance of BRAF expression, we analyzed patient survival in pan-cancer and showed that increased BRAF expression was associated with poor patient overall survival (OS) in LIHC, OV, and UCEC. Interestingly, increased BRAF expression was correlated with better prognosis in BRCA, HNSC, and KIRC (Figure 8A). In addition, survival analysis between BRAF expression and patient relapse-free survival (RFS) across various cancer types exhibited that increased BRAF expression was associated with poor RFS in LIHC, LUSC, and UCEC, while high BRAF expression was correlated with better RFS in BRCA and OV (Figure 8B). The contradictory results in OV may be attributed to insufficient sample size and diverse genetic backgrounds.

## Discussion

In this study, we profiled the characteristics of BRAF in 32 TCGA cancer types by using the cBioPortal tool and the results showed that BRAF expression, methylation, mutations, locations, and CNVs dramatically differed among diverse cancer types, which had significant clinical implications.

DNA methylation, characterized as a methyl group added to cytosines in cytosine-guanine (CpG) dinucleotides, is one of the key epigenetic modifications involved in the regulation of gene expression (Müller and Győrffy, 2022). An existing body of evidence indicates that aberrant DNA methylation leads to activation of oncogenes and silencing of tumor suppressor genes, contributing to tumorigenesis and progression (Klutstein et al., 2016). In a recent study by Noreen et al. (2019), the silencing of Tet methylcytosine dioxygenase 1 (TET1) oxidative DNA demethylase mediated by BRAF V600E mutation was responsible for the initiation of colon cancers with CpG-island methylator phenotype (CIMP). Furthermore, Weisenberger’s group (Weisenberger et al., 2006) also identified the tight association between BRAF mutation and CIMP in colorectal cancers. Our results indicated that the methylation of BRAF and downstream genes were correlated with tumor occurrence. Thus, the potential roles of BRAF mutation in the regulation of DNA methylation and tumor initiation deserve further investigation.

Gene fusions originating from the concatenation of two separate genes caused by trans-splicing events or chromosomal translocations may provide fundamental insights into tumorigenesis and progression (Mertens et al., 2015). In this analysis, the distribution patterns of gene fusions involving BRAF varied in pan-cancer. THCA harbored the highest number of fusion transcripts, of which SND1_BRAF was the most common one, while other cancer types such as LIHC, LUSC, STAD, KIRP, LGG, and BLCA had equally few fusion transcripts. The ectopic expression of SND1_BRAF could increase the phosphorylation levels of MEK/ERK and cell proliferation (Jang et al., 2015). However, MEK inhibitors display expected response rates of up to 70% of patients with higher BRAFV600, while a randomized phase 2 trial showed no differences in overall survival (Algazi et al., 2020). Combination treatments with BRAF fusions and MEK inhibitors may propose a novel insight to evaluate the effectiveness of chemotherapy in cancers.

Mutations in the Pkinase_Tyr domain accounted for most of BRAF single nucleotide or insertion/deletion (indel) mutations. However, the Pkinase_Tyr domain was far more critical in terms of targeted therapy with BRAF inhibitors as approximately 90% of BRAF mutations in SKCM occurred in this region, particularly the BRAF V600E mutation in exon 15. Mutations in this region have been shown to be predictive markers for effective BRAF inhibitors therapy for SKCM in clinical practice (Chapman et al., 2011; McArthur et al., 2014), with significantly longer survival compared to traditional combination chemotherapy. Vemurafenib (PLX4032) and dabrafenib (GSK2118436) were approved by the FDA for the treatment of melanoma patients with BRAF V600E mutation in August 2011 and May 2013, respectively, (Bollag et al., 2012; Hauschild et al., 2012), marking a significant milestone in precision medicine for advanced melanoma. However, due to the complicated pathogenesis of cancer, most patients develop acquired resistance after several months of monotherapy (Shi et al., 2014), combination therapy holds promise as an effective therapeutic strategy. Compared with BRAF inhibitors alone, combining BRAF and MEK inhibitors have been demonstrated to enhance antitumor activity and delay the emergence of drug resistance in patients who have melanoma harboring BRAF V600E mutations, without increased overall toxicity (Robert et al., 2019). In addition, studies assessing triple therapy, BRAF, and MEK inhibitors in combination with immunotherapies, are ongoing (Gutzmer et al., 2020). The BRAF mutation rate of SKCM in this TCGA cohort appeared to be consistent with previous reports and V600E mutations accounted for approximately 95% of all V600 mutated SKCM tumors (Cancer Genome Atlas Network, 2015). For other less frequent BRAF mutations in SKCM, except for the V600K mutation, targeted therapy generated inconsistent results (Menzer et al., 2019). It is clear that different BRAF mutations have different implications, and only those resulting in hyperactivated RAF in MAPK signaling pathway may benefit from BRAF targeted therapy (Yao et al., 2015).

The BRAF V600E mutation in THCA was mainly distributed in papillary thyroid cancer (PTC) and anaplastic thyroid carcinoma (ATC). As previously reported (Chou et al., 2022; Fallahi et al., 2022), PTC and ATC harbored drastically different clinical outcomes while the 5-year survival rate approached 100% in PTC and only 7% in ATC. So, the researches to discover the function of BRAF V600E mutation in ATC are important. Interestingly, the combination therapy of dabrafenib and trametinib was already approved by the FDA in May 2018 for ATC patients with BRAF V600E mutation based on phase II clinical trial (Haddad et al., 2018; Subbiah et al., 2018; Park et al., 2021). Furthermore, the correlation between the BRAF V600E mutation and high-risk clinicopathological features of PTC has been identified in some studies, especially when coexisting with a TERT promoter mutation could remarkably increase transcriptional activities (Liu et al., 2017). As for the PTC patients, studies on BRAF inhibitors have mainly focused on radioactive iodine refractory differentiated thyroid cancer, and relevant clinical trials are in progress (NCT02145143; NCT04462471; NCT04940052; NCT01947023; NCT04554680; NCT05182931).

Although BRAF mutation is an oncogenic driver in multiple cancers, a single-agent BRAF inhibitor has limited clinical efficacy in BRAF V600E-mutated COADREAD patients (Kopetz et al., 2015). This has been attributed predominantly to the rapid reactivation of the MAPK pathway through the epidermal growth factor receptor (EGFR) (Prahallad et al., 2012). Interestingly, combination therapy of BRAF inhibition (encorafenib) and anti-EGFR monoclonal antibodies (cetuximab) has been well validated and approved by FDA in BRAF V600E-mutated metastatic COADREAD patients (BEACON CRC) (Kopetz et al., 2019). Compared with the cetuximab and traditional irinotecan-based chemotherapy, doublet-therapy with encorafenib and cetuximab showed a significant survival benefit, as well as triplet-therapy group with binimetinib (MEK inhibitor). Nowadays, single or combination treatments of encorafenib have been explored in various BRAF mutant cancers, such as SKCM, PAAD, LUAD, COADREAD, THCA, and other advanced solid tumors in a clinical trial (NCT05003622; NCT04390243; NCT05195632; NCT04673955; NCT04061980; NCT03973918). Regarding the expected efficiency of immune checkpoint inhibitors, patients treated with BRAF inhibitors and pembrolizumab or nivolumab are validated in ongoing clinical trials (NCT05217446; NCT04044430; NCT04017650). Although OV and LUAD harbored similar alteration frequencies, the functional profiles of BRAF in these two cancers were quite different, with different amplification and mutation patterns. Studies have shown that BRAF inhibitors alone or in combination with MEK inhibitors are effective as a second-line treatment in patients with BRAFV600-mutated LUAD (Mazieres et al., 2020). BRAF mutation may be a good prognostic factor in OV (Kaldawy et al., 2016), and additional studies will be required to further characterize the clinical significance.

In this study, we profiled BRAF expression, fusion transcript, alteration, and the prognostic and clinical implications across 32 TCGA cancer types. However, some limitations needed to be mentioned. Firstly, some rare tumor types did not have sufficient sample sizes to capture the full BRAF expression and alteration spectrum to establish moderate associations. The low frequency of BRAF mutation or amplification also made this analysis challenging. Moreover, it was mainly a pan-cancer investigation without in-depth dive into each cancer type.

## Conclusion

Our study provides a comprehensive view of BRAF expression, alteration, and clinical prognostic implications across 32 cancer types covering more than ten thousand tumor samples. While some BRAF alternations are involved more in carcinogenesis, others are more therapeutic. Some cancer types have a higher BRAF alternation frequency and its abnormal expression is associated with prognosis. Genomic profiling of BRAF may guide its use in targeted therapy.

## Data Availability

The original contributions presented in the study are included in the article/Supplementary Material, further inquiries can be directed to the corresponding authors.

## References

[B1] Algazi A. P. Othus M. Daud A. I. Lo R. S. Mehnert J. M. Truong T.-G. (2020). Continuous versus Intermittent BRAF and MEK Inhibition in Patients with BRAF-Mutated Melanoma: a Randomized Phase 2 Trial. Nat. Med. 26 (10), 1564–1568. 10.1038/s41591-020-1060-8 33020646 PMC8063889

[B2] Alos L. Fuster C. Castillo P. Jares P. Garcia-Herrera A. Marginet M. (2020). TP53 Mutation and Tumoral PD-L1 Expression Are Associated with Depth of Invasion in Desmoplastic Melanomas. Ann. Transl. Med. 8 (19), 1218. 10.21037/atm-20-1846 33178750 PMC7607103

[B3] Bollag G. Tsai J. Zhang J. Zhang C. Ibrahim P. Nolop K. (2012). Vemurafenib: the First Drug Approved for BRAF-Mutant Cancer. Nat. Rev. Drug Discov. 11 (11), 873–886. 10.1038/nrd3847 23060265

[B4] Cancer Genome Atlas Network (2015). Genomic Classification of Cutaneous Melanoma. Cell 161 (7), 1681–1696. 10.1016/j.cell.2015.05.044 26091043 PMC4580370

[B5] Chapman P. B. Hauschild A. Robert C. Haanen J. B. Ascierto P. Larkin J. (2011). Improved Survival with Vemurafenib in Melanoma with BRAF V600E Mutation. N. Engl. J. Med. 364 (26), 2507–2516. 10.1056/NEJMoa1103782 21639808 PMC3549296

[B6] Chou R. Dana T. Haymart M. Leung A. M. Tufano R. P. Sosa J. A. (2022). Active Surveillance versus Thyroid Surgery for Differentiated Thyroid Cancer: A Systematic Review. Thyroid 32, 351–367. 10.1089/thy.2021.0539 35081743 PMC11265616

[B7] Cremolini C. Moretto R. Zucchelli G. Falcone A. (2019). BRAF Mutant Metastatic Colorectal Cancers: New Arrows in Our Quiver. Ann. Transl. Med. 7 (Suppl. 8), S367. 10.21037/atm.2019.08.118 32016085 PMC6976512

[B8] Fallahi P. Ferrari S. M. Galdiero M. R. Varricchi G. Elia G. Ragusa F. (2022). Molecular Targets of Tyrosine Kinase Inhibitors in Thyroid Cancer. Seminars Cancer Biol. 79, 180–196. 10.1016/j.semcancer.2020.11.013 33249201

[B9] Gao J. Aksoy B. A. Dogrusoz U. Dresdner G. Gross B. Sumer S. O. (2013). Integrative Analysis of Complex Cancer Genomics and Clinical Profiles Using the cBioPortal. Sci. Signal. 6 (269), pl1. 10.1126/scisignal.2004088 23550210 PMC4160307

[B10] Gutzmer R. Stroyakovskiy D. Gogas H. Robert C. Lewis K. Protsenko S. (2020). Atezolizumab, Vemurafenib, and Cobimetinib as First-Line Treatment for Unresectable Advanced BRAF(V600) Mutation-Positive Melanoma (IMspire150): Primary Analysis of the Randomised, Double-Blind, Placebo-Controlled, Phase 3 Trial. Lancet 395 (10240), 1835–1844. 10.1016/S0140-6736(20)30934-X 32534646

[B11] Gyorffy B. (2021). Survival Analysis across the Entire Transcriptome Identifies Biomarkers with the Highest Prognostic Power in Breast Cancer. Comput. Struct. Biotechnol. J. 19, 4101–4109. 10.1016/j.csbj.2021.07.014 34527184 PMC8339292

[B12] Haddad R. I. Nasr C. Bischoff L. Busaidy N. L. Byrd D. Callender G. (2018). NCCN Guidelines Insights: Thyroid Carcinoma, Version 2.2018. J. Natl. Compr. Canc Netw. 16 (12), 1429–1440. 10.6004/jnccn.2018.0089 30545990

[B13] Hauschild A. Grob J.-J. Demidov L. V. Jouary T. Gutzmer R. Millward M. (2012). Dabrafenib in BRAF-Mutated Metastatic Melanoma: a Multicentre, Open-Label, Phase 3 Randomised Controlled Trial. Lancet 380 (9839), 358–365. 10.1016/S0140-6736(12)60868-X 22735384

[B14] Hu X. Wang Q. Tang M. Barthel F. Amin S. Yoshihara K. (2018). TumorFusions: an Integrative Resource for Cancer-Associated Transcript Fusions. Nucleic Acids Res. 46 (D1), D1144–D1149. 10.1093/nar/gkx1018 29099951 PMC5753333

[B15] Jang J. S. Lee A. Li J. Liyanage H. Yang Y. Guo L. (2015). Common Oncogene Mutations and Novel SND1-BRAF Transcript Fusion in Lung Adenocarcinoma from Never Smokers. Sci. Rep. 5, 9755. 10.1038/srep09755 25985019 PMC4434945

[B16] Kaldawy A. Segev Y. Lavie O. Auslender R. Sopik V. Narod S. A. (2016). Low-grade Serous Ovarian Cancer: A Review. Gynecol. Oncol. 143 (2), 433–438. 10.1016/j.ygyno.2016.08.320 27581327

[B17] Karoulia Z. Gavathiotis E. Poulikakos P. I. (2017). New Perspectives for Targeting RAF Kinase in Human Cancer. Nat. Rev. Cancer 17 (11), 676–691. 10.1038/nrc.2017.79 28984291 PMC6000833

[B18] Klutstein M. Nejman D. Greenfield R. Cedar H. (2016). DNA Methylation in Cancer and Aging. Cancer Res. 76 (12), 3446–3450. 10.1158/0008-5472.CAN-15-3278 27256564

[B19] Kopetz S. Desai J. Chan E. Hecht J. R. O'Dwyer P. J. Maru D. (2015). Phase II Pilot Study of Vemurafenib in Patients with Metastatic BRAF-Mutated Colorectal Cancer. J. Clin. Oncol. 33 (34), 4032–4038. 10.1200/JCO.2015.63.2497 26460303 PMC4669589

[B20] Kopetz S. Grothey A. Yaeger R. Van Cutsem E. Desai J. Yoshino T. (2019). Encorafenib, Binimetinib, and Cetuximab in BRAF V600E-Mutated Colorectal Cancer. N. Engl. J. Med. 381 (17), 1632–1643. 10.1056/NEJMoa1908075 31566309

[B21] Kratz J. D. Deming D. A. (2019). The Evolving Treatment Paradigm for BRAF V600 Mutant Colorectal Cancer. Ann. Transl. Med. 7 (Suppl. 8), S257. 10.21037/atm.2019.12.61 32015976 PMC6976492

[B22] Li T. Fu J. Zeng Z. Cohen D. Li J. Chen Q. (2020). TIMER2.0 for Analysis of Tumor-Infiltrating Immune Cells. Nucleic Acids Res. 48 (W1), W509–W514. 10.1093/nar/gkaa407 32442275 PMC7319575

[B23] Liu C.-J. Hu F.-F. Xia M.-X. Han L. Zhang Q. Guo A.-Y. (2018). GSCALite: a Web Server for Gene Set Cancer Analysis. Bioinformatics 34 (21), 3771–3772. 10.1093/bioinformatics/bty411 29790900

[B24] Liu R. Bishop J. Zhu G. Zhang T. Ladenson P. W. Xing M. (2017). Mortality Risk Stratification by Combining BRAF V600E and TERT Promoter Mutations in Papillary Thyroid Cancer: Genetic Duet of BRAF and TERT Promoter Mutations in Thyroid Cancer Mortality. JAMA Oncol. 3 (2), 202–208. 10.1001/jamaoncol.2016.3288 27581851

[B25] Mazieres J. Cropet C. Montané L. Barlesi F. Souquet P. J. Quantin X. (2020). Vemurafenib in Non-small-cell Lung Cancer Patients with BRAF(V600) and BRAF(nonV600) Mutations. Ann. Oncol. 31 (2), 289–294. 10.1016/j.annonc.2019.10.022 31959346

[B26] McArthur G. A. Chapman P. B. Robert C. Larkin J. Haanen J. B. Dummer R. (2014). Safety and Efficacy of Vemurafenib in BRAF(V600E) and BRAF(V600K) Mutation-Positive Melanoma (BRIM-3): Extended Follow-Up of a Phase 3, Randomised, Open-Label Study. Lancet Oncol. 15 (3), 323–332. 10.1016/S1470-2045(14)70012-9 24508103 PMC4382632

[B27] Menzer C. Menzies A. M. Carlino M. S. Reijers I. Groen E. J. Eigentler T. (2019). Targeted Therapy in Advanced Melanoma with Rare BRAF Mutations. J. Clin. Oncol. 37 (33), 3142–3151. 10.1200/JCO.19.00489 31580757 PMC10448865

[B28] Mertens F. Johansson B. Fioretos T. Mitelman F. (2015). The Emerging Complexity of Gene Fusions in Cancer. Nat. Rev. Cancer 15 (6), 371–381. 10.1038/nrc3947 25998716

[B29] Müller D. Győrffy B. (2022). DNA Methylation-Based Diagnostic, Prognostic, and Predictive Biomarkers in Colorectal Cancer. Biochimica Biophysica Acta (BBA) - Rev. Cancer 1877 (3), 188722. 10.1016/j.bbcan.2022.188722 35307512

[B30] Noreen F. Küng T. Tornillo L. Parker H. Silva M. Weis S. (2019). DNA Methylation Instability by BRAF-Mediated TET Silencing and Lifestyle-Exposure Divides Colon Cancer Pathways. Clin. Epigenet 11 (1), 196. 10.1186/s13148-019-0791-1 PMC6916434 31842975

[B31] Palanisamy N. Ateeq B. Kalyana-Sundaram S. Pflueger D. Ramnarayanan K. Shankar S. (2010). Rearrangements of the RAF Kinase Pathway in Prostate Cancer, Gastric Cancer and Melanoma. Nat. Med. 16 (7), 793–798. 10.1038/nm.2166 20526349 PMC2903732

[B32] Park J. Jung H. A. Shim J. H. Park W.-Y. Kim T. H. Lee S.-H. (2021). Multimodal Treatments and Outcomes for Anaplastic Thyroid Cancer before and after Tyrosine Kinase Inhibitor Therapy: a Real-World Experience. Eur. J. Endocrinol. 184 (6), 837–845. 10.1530/EJE-20-1482 33852431

[B33] Planchard D. Smit E. F. Groen H. J. M. Mazieres J. Besse B. Helland Å. (2017). Dabrafenib Plus Trametinib in Patients with Previously Untreated BRAF(V600E)-mutant Metastatic Non-small-cell Lung Cancer: an Open-Label, Phase 2 Trial. Lancet Oncol. 18 (10), 1307–1316. 10.1016/S1470-2045(17)30679-4 28919011

[B34] Prahallad A. Sun C. Huang S. Di Nicolantonio F. Salazar R. Zecchin D. (2012). Unresponsiveness of Colon Cancer to BRAF(V600E) Inhibition through Feedback Activation of EGFR. Nature 483 (7387), 100–103. 10.1038/nature10868 22281684

[B35] Ritterhouse L. L. Barletta J. A. (2015). BRAF V600E Mutation-specific Antibody: A Review. Seminars Diagnostic Pathology 32 (5), 400–408. 10.1053/j.semdp.2015.02.010 25744437

[B36] Robert C. Grob J. J. Stroyakovskiy D. Karaszewska B. Hauschild A. Levchenko E. (2019). Five-Year Outcomes with Dabrafenib Plus Trametinib in Metastatic Melanoma. N. Engl. J. Med. 381 (7), 626–636. 10.1056/NEJMoa1904059 31166680

[B37] Samatar A. A. Poulikakos P. I. (2014). Targeting RAS-ERK Signalling in Cancer: Promises and Challenges. Nat. Rev. Drug Discov. 13 (12), 928–942. 10.1038/nrd4281 25435214

[B38] Shi H. Hugo W. Kong X. Hong A. Koya R. C. Moriceau G. (2014). Acquired Resistance and Clonal Evolution in Melanoma during BRAF Inhibitor Therapy. Cancer Discov. 4 (1), 80–93. 10.1158/2159-8290.CD-13-0642 24265155 PMC3936420

[B39] Song L.-B. Zhang Q.-J. Hou X.-Y. Xiu Y.-Y. Chen L. Song N.-H. (2020). A Twelve-Gene Signature for Survival Prediction in Malignant Melanoma Patients. Ann. Transl. Med. 8 (6), 312. 10.21037/atm.2020.02.132 32355756 PMC7186619

[B40] Subbiah V. Kreitman R. J. Wainberg Z. A. Cho J. Y. Schellens J. H. M. Soria J. C. (2018). Dabrafenib and Trametinib Treatment in Patients with Locally Advanced or Metastatic BRAF V600-Mutant Anaplastic Thyroid Cancer. J. Clin. Oncol. 36 (1), 7–13. 10.1200/JCO.2017.73.6785 29072975 PMC5791845

[B41] Tang Z. Kang B. Li C. Chen T. Zhang Z. (2019). GEPIA2: an Enhanced Web Server for Large-Scale Expression Profiling and Interactive Analysis. Nucleic Acids Res. 47 (W1), W556–W560. 10.1093/nar/gkz430 31114875 PMC6602440

[B42] Wang Q. Zhao N. Zhang J. (2021). Gene Mutation Analysis in Papillary Thyroid Carcinoma Using a Multi-Gene Panel in China. Int. J. Gen. Med. 14, 5139–5148. 10.2147/IJGM.S327409 34511996 PMC8421255

[B43] Wang T.-X. Tan W.-L. Huang J.-C. Cui Z.-F. Liang R.-D. Li Q.-C. (2020). Identification of Aberrantly Methylated Differentially Expressed Genes Targeted by Differentially Expressed miRNA in Osteosarcoma. Ann. Transl. Med. 8 (6), 373. 10.21037/atm.2020.02.74 32355817 PMC7186728

[B44] Weisenberger D. J. Siegmund K. D. Campan M. Young J. Long T. I. Faasse M. A. (2006). CpG Island Methylator Phenotype Underlies Sporadic Microsatellite Instability and Is Tightly Associated with BRAF Mutation in Colorectal Cancer. Nat. Genet. 38 (7), 787–793. 10.1038/ng1834 16804544

[B45] Xu M. Zhou J. Zhang Q. Le K. Xi Z. Yi P. (2020). MiR-3121-3p Promotes Tumor Invasion and Metastasis by Suppressing Rap1GAP in Papillary Thyroid Cancer *In Vitro* . Ann. Transl. Med. 8 (19), 1229. 10.21037/atm-20-4469 33178761 PMC7607113

[B46] Yao Z. Torres N. M. Tao A. Gao Y. Luo L. Li Q. (2015). BRAF Mutants Evade ERK-dependent Feedback by Different Mechanisms that Determine Their Sensitivity to Pharmacologic Inhibition. Cancer Cell 28 (3), 370–383. 10.1016/j.ccell.2015.08.001 26343582 PMC4894664

[B47] Zhang R. Li Y. Yu H. Liu L. Zhu C. Zuo S. (2020). An Aberrant DNA Methylation Signature for Predicting Hepatocellular Carcinoma. Ann. Transl. Med. 8 (24), 1667. 10.21037/atm-20-7804 33490179 PMC7812168

[B48] Zhao R. Gao S. He H. Zhang J. Zhang G. Wen X. (2021). Evaluation on the Distribution of EGFR, KRAS and BRAF Genes and the Expression of PD-L1 in Different Types of Lung Cancer. Int. J. Gen. Med. 14, 5615–5620. 10.2147/IJGM.S316151 34548813 PMC8449634

