# Supplementary material for: Spectrum of BRAF Aberrations and Its Potential Clinical Implications: Insights From Integrative Pan-Cancer Analysis
Source: Front Bioeng Biotechnol. 2022 Jul 14;10:806851. doi: 10.3389/fbioe.2022.806851 (PMC9329936; doi:10.3389/fbioe.2022.806851)
Supplement: Supplementary file 2 [file Table1.DOCX]

Supplementary Table S1. The main bioinformatics tools used to analyze the role of BRAF in pan-cancer.

| Database | URL | References |
| --- | --- | --- |
| cBioPortal | <http://cbioportal.org/> | (Hu et al., 2018) |
| TIMER2 | <http://timer.cistrome.org/> | (Li et al., 2020) |
| GEPIA2 | <http://gepia.cancer-pku.cn/> | (Tang et al., 2019) |
| GSCALite | <http://bioinfo.life.hust.edu.cn/web/GSCALite/> | (Liu et al., 2018) |
| Kaplan-Meier Plotter | <http://kmplot.com/> | (Gyorffy, 2021) |
