# Supplementary figures and images for: Spectrum of BRAF Aberrations and Its Potential Clinical Implications: Insights From Integrative Pan-Cancer Analysis
Source: Front Bioeng Biotechnol. 2022 Jul 14;10:806851. doi: 10.3389/fbioe.2022.806851 (PMC9329936; doi:10.3389/fbioe.2022.806851)

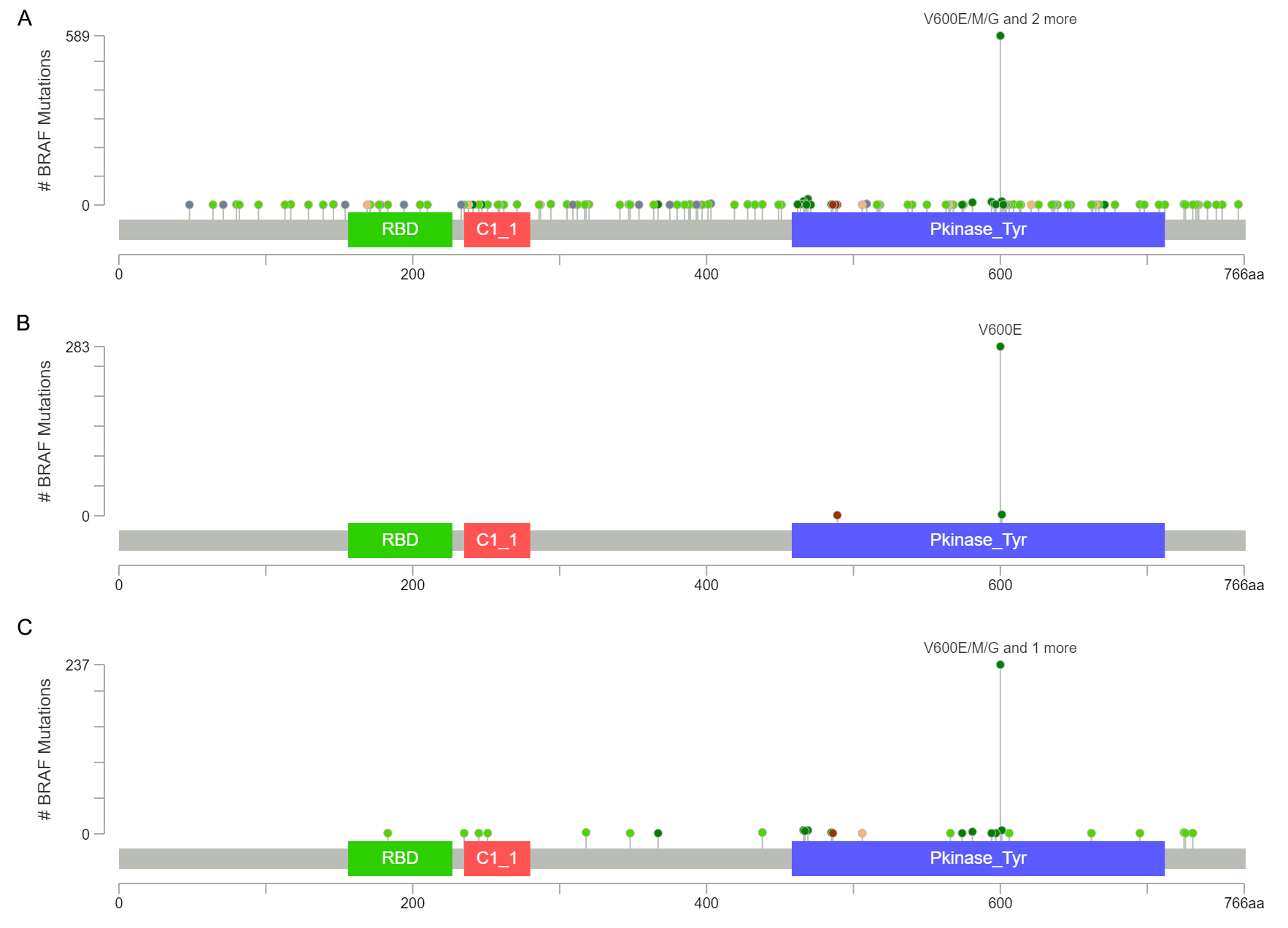

Supplement: Supplementary file 3 [file Image1.JPEG]

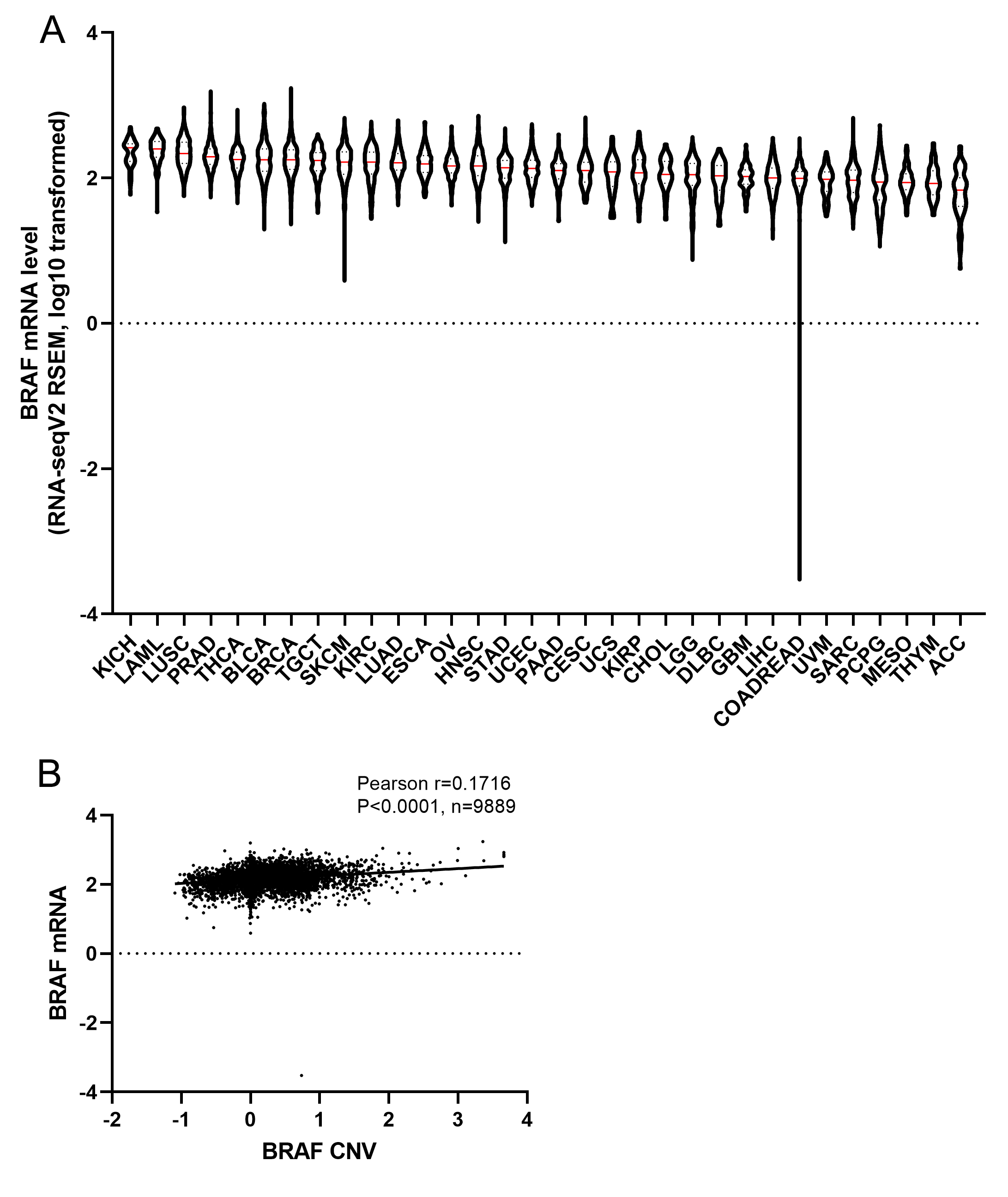

Supplement: Supplementary file 4 [file Image2.JPEG]
