# Supplementary material for: Spectrum of BRAF Aberrations and Its Potential Clinical Implications: Insights From Integrative Pan-Cancer Analysis
Source: Front Bioeng Biotechnol. 2022 Jul 14;10:806851. doi: 10.3389/fbioe.2022.806851 (PMC9329936; doi:10.3389/fbioe.2022.806851)
Supplement: Supplementary file 6 [file Table3.DOCX]

Supplementary Table S3. Summary of TCGA cancer types and sample size.

| Cancer Abbreviation | Cancer Type | Sample Size |
| --- | --- | --- |
| ACC | Adrenocortical carcinoma | 92 |
| BLCA | Bladder urothelial carcinoma | 411 |
| BRCA | Breast invasive carcinoma | 1084 |
| CESC | Cervical squamous cell carcinoma and endocervical adenocarcinoma | 297 |
| CHOL | Cholangiocarcinoma | 36 |
| COADREAD | Colon adenocarcinoma /Rectum adenocarcinoma | 594 |
| DLBC | Lymphoid neoplasm diffuse large B-cell lymphoma | 48 |
| ESCA | Esophageal carcinoma | 182 |
| GBM | Glioblastoma multiforme | 585 |
| HNSC | Head and Neck squamous cell carcinoma | 523 |
| KICH | Kidney chromophobe | 65 |
| KIRC | Kidney renal clear cell carcinoma | 512 |
| KIRP | Kidney renal papillary cell carcinoma | 283 |
| LAML | Acute myeloid leukemia | 200 |
| LGG | Brain lower grade glioma | 514 |
| LIHC | Liver hepatocellular carcinoma | 372 |
| LUAD | Lung adenocarcinoma | 566 |
| LUSC | Lung squamous cell carcinoma | 487 |
| MESO | Mesothelioma | 87 |
| OV | Ovarian serous cystadenocarcinoma | 585 |
| PAAD | Pancreatic adenocarcinoma | 184 |
| PCPG | Pheochromocytoma and Paraganglioma | 178 |
| PRAD | Prostate adenocarcinoma | 494 |
| SARC | Sarcoma | 255 |
| SKCM | Skin cutaneous melanoma | 442 |
| STAD | Stomach adenocarcinoma | 440 |
| TGCT | Testicular germ cell tumors | 149 |
| THCA | Thyroid carcinoma | 499 |
| THYM | Thymoma | 123 |
| UCEC | Uterine corpus endometrial carcinoma | 529 |
| UCS | Uterine carcinosarcoma | 57 |
| UVM | Uveal Melanoma | 80 |
